# Noncoding RNAs in tumorigenesis and tumor therapy

**DOI:** 10.1016/j.fmre.2023.05.014

**Published:** 2023-06-12

**Authors:** Pingping Zhu, Benyu Liu, Zusen Fan

**Affiliations:** a CAS Key Laboratory of Infection and Immunity, CAS Center for Excellence in Biomacromolecules, Institute of Biophysics, Chinese Academy of Sciences, Beijing 100101, China; b School of Life Sciences, Zhengzhou University, Zhengzhou 450001, China; c Research Center of Basic Medicine, Academy of Medical Sciences, Zhengzhou University, Zhengzhou 450001, China

**Keywords:** LncRNA, CircRNA, Cancer stem cells, Tumorigenesis, Niche

## Abstract

Tumorigenesis is a complicated process in which numerous modulators are involved in different ways. Previous studies have focused primarily on tumor-associated protein-coding genes such as oncogenes and tumor suppressor genes, as well as their associated oncogenic pathways. However, noncoding RNAs (ncRNAs), rising stars in diverse physiological and pathological processes, have recently emerged as additional modulators in tumorigenesis. In this review, we focus on two typical kinds of ncRNAs: long noncoding RNAs (lncRNAs) and circular RNAs (circRNAs). We describe the molecular patterns of ncRNAs and focus on the roles of ncRNAs in cancer stem cells (CSCs), tumor cells, and tumor environmental cells. CSCs are a small subset of tumor cells and are generally considered to be cells that initiate tumorigenesis, and dozens of ncRNAs have been defined as critical modulators in CSC maintenance and oncogenesis. Moreover, ncRNAs are widely involved in oncogenetic processes, including sustaining proliferation, resisting cell death, genome instability, metabolic disorders, immune escape and metastasis. We also discuss the potential applications of ncRNAs in tumor diagnosis and therapy. The progress in ncRNA research greatly improves our understanding of ncRNAs in oncogenesis and provides new potential targets for future tumor therapy.

## 1 Introduction

Tumors are initiated by cancer stem cells (CSCs) [1] and feature various hallmarks, including aberrant cell proliferation, evasion of growth suppression, and tumor immune escape [2,3]. With advances in next-generation sequencing and applications of bioinformatics in tumor biology in recent years, there has been a rapid boost in our understanding of tumorigenesis, especially at the molecular and genetic levels [4]. The role of functional proteins, encoded by oncogenes and tumor repressor genes, in tumor initiation and maintenance has been investigated extensively, while RNAs have become the focus of cancer research during the last two decades and have deeply changed our understanding of tumorigenesis and progression, as well as provided important clues for tumor diagnosis and therapy [5].

RNA molecules are intermediate bridges in the transmission of genetic information from DNA to protein, while massive RNAs without protein-coding potential have been discovered and defined as noncoding RNAs (ncRNAs) [6]. In addition to general housekeeping functions such as ribosome RNAs (rRNAs), transfer RNA (tRNAs), small nuclear RNAs (snRNA) and small nucleolar RNAs (snoRNAs), the regulatory functions of ncRNAs have emerged in the last two decades, including microRNAs (miRNAs), long noncoding RNAs (lncRNAs), circular RNAs (circRNAs) and PIWI-interacting RNAs (piRNAs) [7]. In the last two decades, accumulating ncRNAs have been identified as modulators in tumor initiation and tumor hallmark maintenance [8].

In this review, we focus on tumorigenesis and tumor invention regulated by lncRNAs and circRNAs, two typical types of ncRNAs. We discuss recent progress in understanding the molecular patterns of ncRNAs and the regulatory roles of ncRNAs in tumorigenesis and define functional ncRNAs in CSC self-renewal, tumor hallmark maintenance, niche regulation, and therapeutic applications.

## 2 Noncoding RNAs

### 2.1 Classification of ncRNA

LncRNAs, characterized by an arbitrary length (>200 nucleotides) and lack of protein-coding capacity, are well-known modulators in various physiological and pathological processes. According to the relative genomic location of lncRNAs and their neighboring protein-coding genes, lncRNAs can be grouped into intergenic lncRNAs, intronic lncRNAs, sense lncRNAs, and antisense lncRNAs [9]. LncRNAs were discovered in the early 1990s and over 100 thousand human lncRNAs have been identified.

CircRNAs are circular transcripts, which are generated through covalent conjugation of 5’ and 3’ ends through back splicing [10]. The unique circular architecture of circRNAs confers the resistance to exonuclease and thus they are considered relative stable transcripts. CircRNAs were discovered in 1976 and were traditionally regarded as the byproducts of RNA polymerase II, but considered one of central ncRNAs in recent years [11].

Based on the advancement of high-throughput RNA sequencing, mass spectrometry, CRISPR/Cas9, as well as the involvement of bioinformatic analysis and bioorthogonal chemistry, accumulative novel strategies for noncoding RNA research have been exploited, and many new kinds or new functions of noncoding RNAs have been defined, largely extending the repertories of ncRNAs. One of the most surprising discoveries is the existence of peptide-coding ncRNAs, including peptide-coding lncRNAs and peptide-coding circRNAs, which generate peptides via IRES (internal ribosome entry site)-mediated or m^6^A-mediated cap-independent translation [12]. For example, myoregulin and DWORF (dwarf open reading frame), two micropeptides involved in muscle performance and calcium pump, are coded by previously annotated lncRNAs LINC00948 and LINC00961, respectively [13,14]. CircMbl and circ-ZNF609 harbor protein-coding potentials in a cap-independent manner [15,16]. Owing to advanced technologies and sequencing methods, accumulating ncRNAs with coding potential have been identified [17].

Chimeric genes, originating from more than one gene locus through fusion (distant genes) or read-through (adjacent genes), are widespread phenomena. Correspondingly, chimeric ncRNAs, including fusion-lncRNAs, fusion-circRNAs, read-through lncRNAs, and read-through circRNAs, have been discovered [18], [19], [20]. It is predictable that large numbers of chimeric ncRNAs will be identified with the development of RNA-sequencing technology.

Besides the nucleus, the mitochondrion is another organelle that harbors DNA and transcription process, generating mitochondrial-lncRNAs and mitochondrial-circRNAs. Several mitochondrial DNA encoded circular RNAs (mecciRNAs) have been defined and mcPGK1 interacts with TOM40 complex to facilitate the mitochondrial importation of nucleus-encoded protein PGK1 [21]. Steatohepatitis- associated circRNA ATP5B Regulator (SCAR), another mitochondria-encoded circRNA, binds to ATP5B directly to close mPTP (mitochondrial permeability transition pore), subsequently inhibits the output of mitochondrial ROS and finally prevents the hyperactivation of NASH fibroblasts [22].

Extracellular RNAs (exRNAs, or ecRNAs) are RNA species present in extracellular vesicles, such as exosomes, microvesicles, and oncosomes. Abundant ncRNAs, including microRNAs, lncRNAs, and circRNAs, are present in these particles and probably function in intercellular communications. The characteristics of abundant exRNAs in blood, saliva, breast milk, and urine open new strategies for liquid biopsy, and circRNAs are promising biomarkers because of their high stability [23,24].

### 2.2 Regulatory mechanisms of ncRNAs

**Fig. 1:**
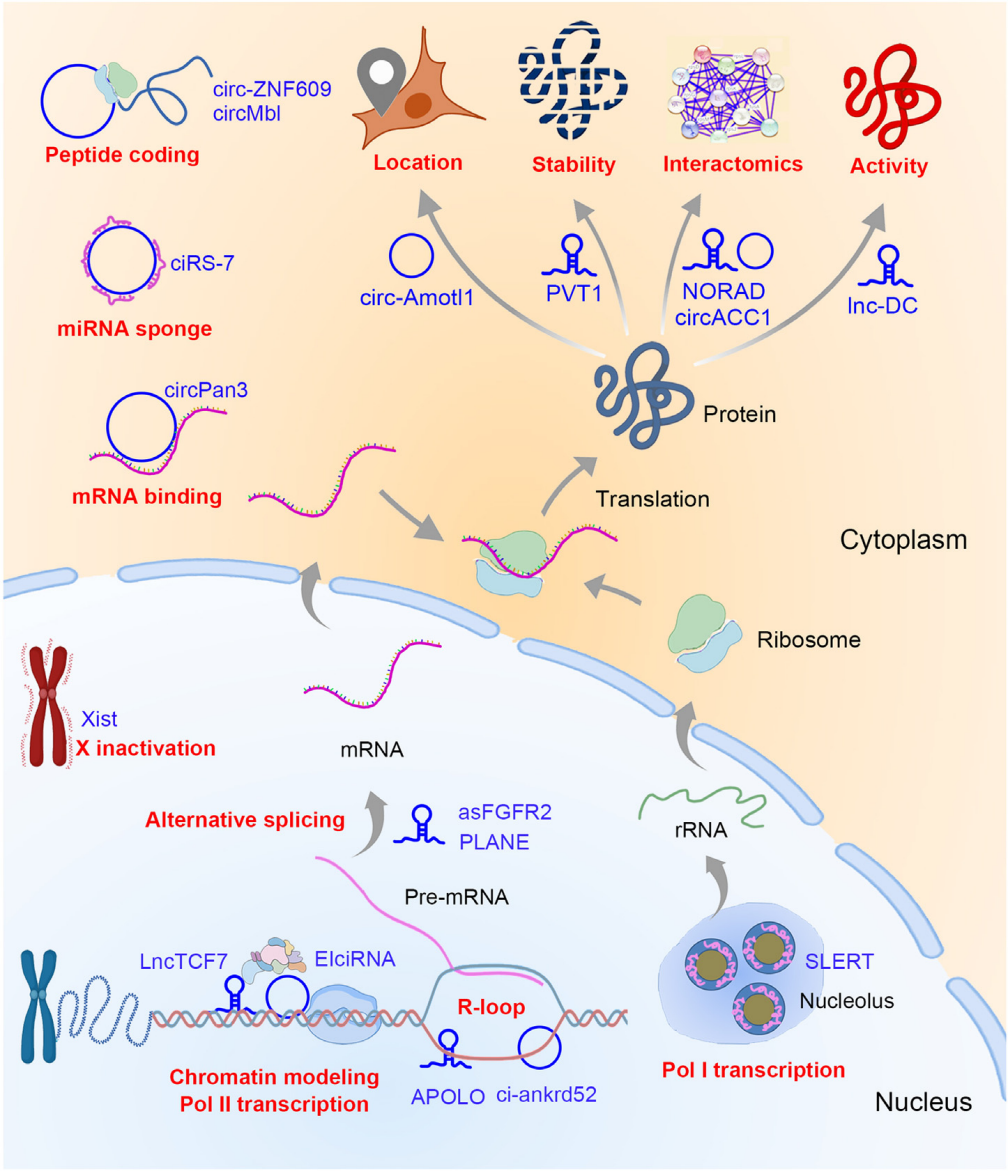
**Regulatory mechanism of ncRNAs.** ncRNAs exert their regulatory role at DNA, RNA, and protein levels. At the DNA level, ncRNAs are involved in X inactivation, chromatin modeling, polymerase II (Pol II) transcription, R-loop formation, and Pol I transcription. At the RNA level, ncRNAs are involved in alternative splicing, mRNA decay and translation as miRNA sponges or mRNA binding partners. At the protein level, ncRNAs are involved in peptide coding, protein location, stability, interactomics, and activity regulation. Typical ncRNAs are denoted as blue words and regulatory patterns are denoted as red words.

LncRNAs and circRNAs function through various mechanisms, involving chromatin accessibility, transcription, RNA processing and stability, translocation, protein stability, and functionality (Fig. 1).

The accessibility of chromatin is a fundamental requisite for transcriptional activation. Numerous lncRNAs bind to one strand of the DNA helix to form DNA-RNA chimeras (R-loops), and regulate the transcriptional activation of target genes [25]. Circular intronic RNAs harbor a stronger ability of R-loop formation compared to the corresponding pre-mRNAs and are then degraded with RNase H1 in an R-loop-dependent manner. This “replacement and cleavage” model of circular intronic RNAs inhibits the formation of pre-mRNA R-loops and facilitates the release of pre-mRNA for efficient transcription elongation. As an example, circRNA ci-ankrd52 promoted transcriptional elongation by facilitating the release of *Ankrd52* pre-mRNA from the R-loop [26]. LncRNA Xist (X Inactive Specific Transcript), a major effector of the X-inactivation process, combined with X chromatin to inhibit overall accessibility and induce H3K27me3 via recruiting PRC2 [27].

RNA-RNA interactions between lncRNAs/circRNAs and other ncRNAs or pre-mRNAs extend the regulatory layers of lncRNAs/circRNAs. MicroRNAs are endogenously generated single stranded ncRNAs that interact with target mRNAs via sequence-complementarity, subsequently triggering the degradation or modification of target transcripts. Serving as typical competing endogenous RNAs (ceRNAs), some circRNAs can sponge and suppress the activities of microRNAs [28]. With over 70 miR-7 binding sites, ciRS-7 (circular RNA sponge for miR-7) shows a strong capacity to sponge and suppress miR-7 activity and thus increase miR-7 target gene expression [29]. However, only limited ncRNAs can be served as miRNA sponges since many ncRNAs do not contain multiple miRNA-binding sites according to bioinformatic analysis. EIciRNA (Exon-intron circular RNAs) interacts with U1 snRNA, another kind of ncRNA, to form EIciRNA–U1 snRNP complex, which further recruits RNA polymerase II at the promoter regions of parent genes to enhance their transcription, rather than RNA processing that is the conventional function of U1 snRNA [30]. Besides combining with miRNAs or snRNAs, lncRNAs and circRNAs can interact with premature or mature mRNAs to modulate their processing, nuclear export, decay, or translation. LncRNA PLANE (Pan-cancer LncRNA Activating NCOR2 responsive to E2F1) is up-regulated by copy number gain and E2F1-driven transcription activation, and then combines with *NCOR2* pre-mRNA to modulate its alternative splicing, finally drives tumorigenesis and tumor propagation in various tumor types [31]. LncRNA asFGFR2 (antisense FGFR2 transcript) also modulates the alternative splicing of *FGFR2* pre-mRNA via recruiting PRC2 and KDM2a, which establishes chromatin signatures for mesenchymal-specific splicing [32]. CircPan3, a circRNA highly expressed in intestinal stem cells, interacts with *Il13Ra1* mRNA via sequence-complementarity and acts as a ceRNA to inhibit the combination between *Il13Ra1* mRNA and Ksrp, a critical inducer for mRNA decay. Accordingly, circPan3 maintains *Il13ra1* mRNA stability and drives the expression of surface protein Il13ra1, subsequently modulating the self-renewal of intestinal stem cells [33].

More than 1500 RNA-binding proteins (RBPs) in cells have been identified, which exert various biological functions [34]. The interaction between ncRNAs and RBPs regulates their subcellular locations, stabilities, interactomics, conformations, and activities. Circ-Amotl1 is robustly expressed in several kinds of cancer cells and exerts an oncogenic role through interaction with c-Myc, a key oncogene in various tumor types. Circ-Amotl1 is located in the nucleus and drives the nuclear retention of c-Myc, which is required for c-Myc transcriptional activity [35]. Another c-Myc-binding ncRNA is PVT-1, which was cloned in the 1980s as one of the earliest cloned lncRNAs and emerges as a rising star lncRNA recently. Both PVT-1 and c-Myc originate from 8q24.21 chromosomal region and are frequently copy-number gained in many human cancers. Copy number gains induce a robust expression of PVT-1, which enhances the stability of c-Myc through direct “PVT1-c-Myc” interaction [36]. NORAD (non-coding RNA activated by DNA damage) harbors a strong RBMX-binding potential and controls the ability of RBMX to assemble the ribonucleoprotein complex NARC1 (NORAD-activated ribonucleoprotein complex 1), which contains several suppressors of genomic instability and is involved in genomic stability maintenance. NORAD is required for the assembly of NARC1, providing a paradigm of lncRNA in controlling RBPs to form a higher-order complex [37]. Nucleolar-locating lncRNA SLERT (snoRNA-ended long noncoding RNA that enhances pre-rRNA transcription) is required for the maintenance of closed conformation of its interacting protein DDX21 and drives the transitions from multimer to monomer DDX21, which loosens the DDX21 ring and drives liquid-liquid phase separation of FC/DFC, finally promotes the Pol I accessibility and ribosomal RNA production [38,39]. Lnc-DC is exclusively expressed lncRNA in conventional dendritic cells (DC) and promotes the maturation and T cell-stimulating function of DC through activation of STAT3 (signal transducer and activator of transcription 3) directly. Lnc-DC binds to STAT3 to prevent the interaction of STAT3 and SHP1 which inactivates STAT3 via dephosphorylation of STAT3 on tyrosine-705 [40].

With a deep understanding of the functions of lncRNAs and circRNAs, many reviews about lncRNAs and circRNAs have been published, and readers can refer to these reviews for details [9,11]. Herein, we focus on the involvement of noncoding RNAs, specifically lncRNAs and circRNAs, in the regulation of tumorigenesis, including cancer stem cells, cancer cells, and niche cells.

## 3 Regulation of ncRNAs in cancer stem cell

### 3.1 Cancer stem cells

Heterogeneity is the basic characteristic of tumors, and almost all tumors contain diverse kinds of tumor cells. Among them, cancer stem cells (CSCs) attract the most attention. CSCs are a small subset of cells within the tumor bulk that harbor self-renewal and differentiation capacities, and these cells drive tumor initiation, drug resistance, relapse, and metastasis. Many markers have been identified for various CSCs, and CD133 and CD13 are frequently identified as CSC markers for many tumor types [41,42]. Many CSC markers are target genes of CSC-activating signaling pathways, including the Wnt/β-catenin, Notch, and Hedgehog signaling pathways, and targeting these signaling pathways holds promise for clinical outcomes [43]. Applications of single-cell sequencing, spatial transcriptomics, and lineage tracing largely facilitate studies of CSC identification, characteristics, and functionality.

Cancer stem cells harbor the properties of cancer cells and stem cells simultaneously, and several typical stemness factors have been proven to modulate CSC maintenance, including c-Myc, Sox2, and Sox4 [44]. Zhu et al. identified functional genes in liver CSC self-renewal via screening genes that are abnormally expressed in liver cancer cells and stem cells simultaneously, and revealed C8orf4 and Zic2 as modulators in liver CSC self-renewal. C8orf4 is lowly expressed in liver cancer cells and embryonic stem cells and inhibits the stemness of liver CSCs by blocking the activation of Notch2 signaling. C8orf4 interacts with and promotes the cytoplasmic retention of Notch2, and thus blocks the transcription of Notch target genes Hey1 and Nrarp, which are required for liver CSC self-renewal [45]. On the contrary, Zic2 is highly expressed in liver cancer and embryonic stem cells and drives the self-renewal of liver CSCs through directly targeting Oct4. Zic2 binds to *Oct4* promoter and interacts with Rbbp4, a component of NURF (nucleosome remodeling factor) complex, which eventuallypromoting the transcriptional activation of Oct4 [46].

The epigenetic disorder is a typical hallmark of CSCs, and chromatin remodeling complexes are involved in the epigenetic regulation of stemness factors. Several components of chromatin remodeling complexes are frequently mutated, including the SWI/SNF (Switch defective/sucrose non-fermentable) components ARID1A and SMARCA4 (BRG1) and the PRC component EZH2 [47,48]. Overexpression and hyperactivation of chromatin remodeling complexes, including SWI/SNF complex, NURD complex, NURF complex, PRC complex, and TIP60/P400 complex, are common landscapes in various cancers. SMARCA2 (BRM) and SMARCA4 (BRG1) are mutually exclusionary in SWI/SNF complex, forming BRM-type SWI/SNF and BRG-type SWI/SNF. Zhu et al. revealed an increased expression of BRG1 that enhances the assembly of BRG-type SWI/SNF complex in liver CSCs, on the contrary, BRM and BRM-type SWI/SNF are decreased. Interestingly, a BRM-interacting lncRNA highly expressed in liver CSCs, termed lncBRM, modulates the switch from BRG-type SWI/SNF to BRM-type SWI/SNF [49].

CSCs reside in a complicated microenvironment composed of various niche cells and niche factors, which provide biophysicochemical cues to maintain the survival, self-renewal, differentiation, and functionalities of CSCs [50]. Recently, accumulating niche cells and niche factors have been identified for CSCs, including mesenchymal cells, immune cells, and neurons [51]. Zhu et al. identified a cross-talk factor between colorectal neurons and colorectal CSCs. By engaging 5-HT secreted by niche neurons, colorectal CSCs show enhanced activation of the Wnt (Wingless/Integrated) /β-catenin signaling pathway and a stronger capacity for self-renewal and metastasis [52]. Moreover, secretion of 5-HT by neurons is further regulated by isovalerate, a metabolite produced by colorectal cancer-enriched microbiota, mimicking the “microbiota-neuron” regulatory mechanism under physiological conditions [53].

**Fig. 2:**
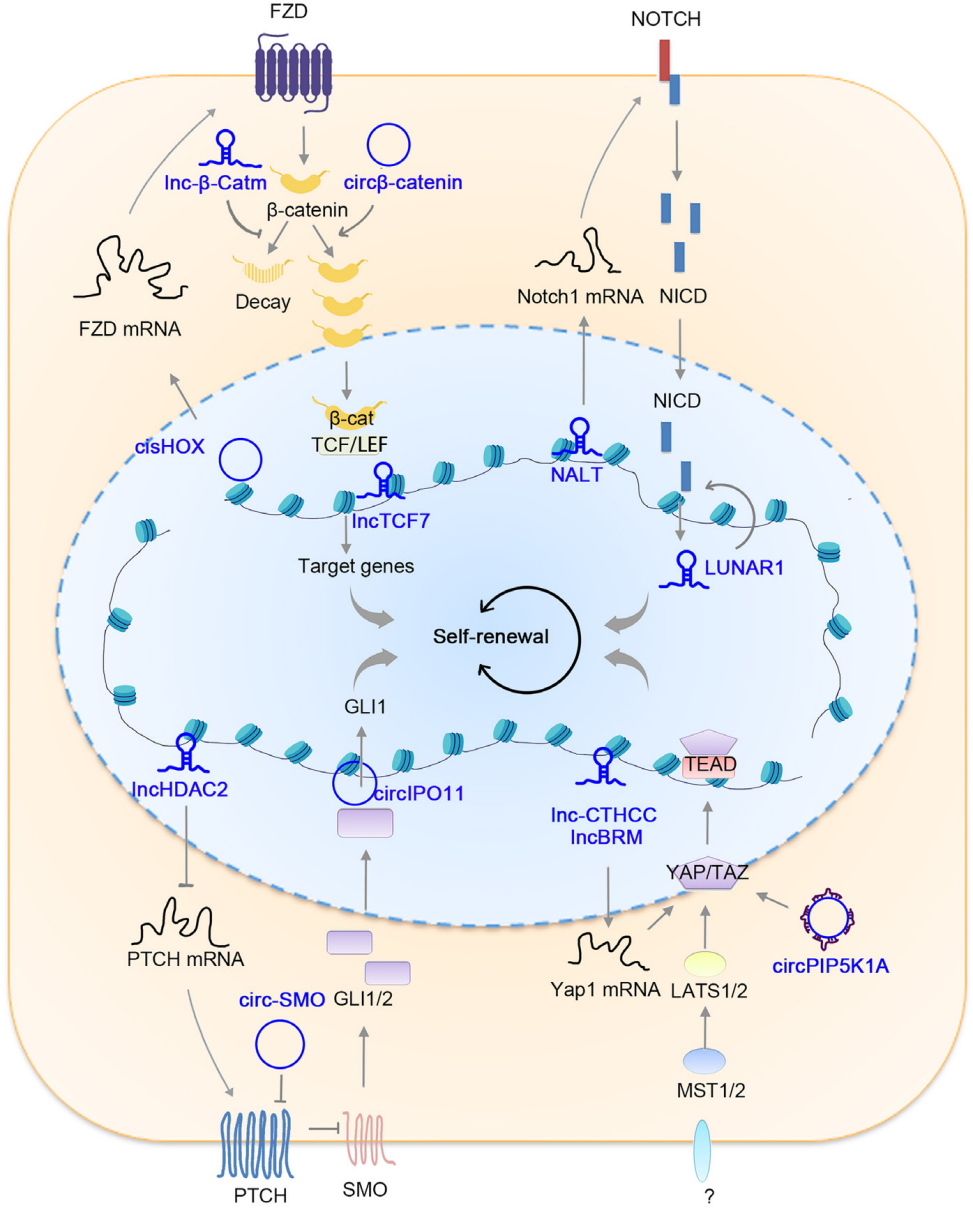
**ncRNAs in CSC regulation.** The Wnt/β-catenin, Notch, Hedgehog (Hh) and Hippo/Yap signaling pathways are the most important pathways in CSC regulation, and their associated ncRNAs are shown. For Wnt/β-catenin regulation, cisHOX induces FZD3 expression, lnc-β-catm and circβ-catenin are involved in β-catenin stability, and lncTCF7 promotes the expression of Wnt target gene TCF7. For Notch regulation, NALT promotes Notch1 transcription, and LUNAR1 drives the expression of various Notch target genes. For Hh regulation, lncHDAC2 promotes Hh activation by repressing PTCH expression, whereas circIPO11 induces the expression of the Hh target gene GLI1. For Hippo/Yap regulation, lnc-CTHCC and lncBRM promote Yap1 transcription, while circPIP5K1A drives Yap1 activation as miR-515-5p sponge.

Overall, CSCs exert fundamental functions in tumorigenesis and are regulated by various intrinsic stem factors, epigenetic factors, and niche factors. The relationship between CSCs and tumorigenesis has been discussed previously [54],while we focus on the involvement of ncRNAs in the regulation of CSCs, including transcription factors (TFs), Wnt pathway, Notch pathway, Hedgehog pathway, Yap pathway and other pathways (Fig. 2) in this section.

### 3.2 Transcription factors

Transcription factors play critical roles in cell fate decisions, and the identification and functionality of CSCs are surely regulated by transcription factors [55]. As a key TF in tumorigenesis and stemness regulation, c-Myc is posttranscriptionally regulated by its adjacent lncRNA PVT1, whose copy number is increased synchronously with that of c-Myc and required for increased c-Myc protein levels by maintaining protein stability [36]. The nuclear translocation of c-Myc, a requisite process for c-Myc function, is regulated by its interacting circRNA circ-Amotl1 [35]. Wang et al. identified lncTCF7 as a lncRNA modulator of TCF7, which is a typical transcription factor in T lymphocyte development and has recently emerged as a tumor suppressor or tumor enhancer [56]. The lncTCF7 is highly expressed in liver cancer and liver CSCs, recruits the SWI/SNF complex to the promoter of an adjacent gene TCF7 and promotes TCF7 expression *in cis*, finally driving Wnt signaling activation and liver CSC self-renewal [56].

Besides the regulatory role of ncRNAs in stemness TFs, some ncRNAs exert their roles via ordinary TFs. MAFF (MAF bZIP transcription factor F), a member of MAF family of TFs, is regulated by circular RNA cia-MAF at transcriptional level. The cia-MAF is robustly expressed in liver cancer and liver CSCs, and is required for liver CSC self-renewal and tumorigenesis via loss of function (LOF) and ectopic expression assays. A cia-MAF binds to *MAFF* promoter and recruits TIP60/P400 complex to *MAFF* promoter region, and induces MAFF expression via a TIP60/P400-dependent manner. MAFF has a critical role in liver CSC self-renewal and metastasis and may serve as a targeting candidate for CSCs without MAFA/MAFG-copy number gain, whereas in CSCs with MAFA/MAFG-copy number gain, MAFF isn't highest expressed MAF TFs and other TFs still work even when MAFF is blocked [57]. LncGata6 is highly expressed in intestinal stem cells, colorectal tumors, and colorectal CSCs, and is required for colorectal tumor propagation and the maintenance of colorectal CSCs. LncGata6 depletion largely diminishes tumor initiation and progression both in humans and mice. LncGata6 combines with *Ehf* promoter and recruits NURF chromatin remodeling complex to *Ehf* promoter to drive its transcription. Ehf, in turn, activates Wnt/β-catenin signaling pathway via promoting the expression of Lgr4 and Lgr5, which are Rspo receptors and conjugated with Rspo ligands produced from niche cells. Through the NURF-Ehf-Lgr4/5-Wnt axis, lncGata6 exerts its role in colorectal stem cell self-renewal [58]. Different from the transcriptional regulatory roles of cia-MAF and lncGata6, circRNA cis-HOX enhances the mRNA stability of another TF HOXC10. cis-HOX is highly expressed in colorectal CSCs, binds to *HOXC10* mRNA via sequence-complementarity, and functions in a ceRNA-like manner to compete for the combination between *HOXC10* mRNA and Ksrp, a major component for mRNA decay. By regulating mRNA stability, cis-HOX promotes HOXC10 expression, which further induces the expression of FZD3 (frizzled class receptor 3), a Wnt ligand with the highest expression in colorectal CSCs. Moreover, HOXC10 inhibitor salinomycin shows an optimal therapeutic potential for colorectal CSCs without *APC* mutation, whereas in APC mutate CSCs, Wnt/β-catenin signaling is active constitutively and cannot be regulated by modulators as upstream of APC [59].

### 3.3 Wnt/β-catenin signaling

Wnt/β-catenin signaling is the most important signaling pathway in CSCs from various tumor types [60]. CSCs express FZD family proteins on the cell membrane, which are Wnt receptors. Upon engagement of extracellular Wnt ligands, the APC complex responsible for β-catenin degradation, is disrupted, and thus, β-catenin is accumulated and promotes the expression of Wnt/β-catenin target genes, including c-Myc, Axin2, Ccnd1, TCF7, Lgr5 and so on [61]. These target genes are involved in various physiological and pathological processes, especially in tumorigenesis and CSC self-renewal. NcRNAs modulate Wnt/β-catenin activation at various levels, including FZD expression, β-catenin expression, modification, stability, and target gene expression.

It's well known that the activation of Wnt/β-catenin signaling is required for CSC self-renewal, while the regulatory mechanism of Wnt/β-catenin activation is less investigated. In colon cancer and CSCs, FZD3 is identified as the highest expressed FZDs and required for Wnt/β-catenin activation. Moreover, FZD3 expression is driven by cis-HOX-dependent HOXC10 activation [59]. Whereas in liver tumorigenesis and liver CSCs, FZD6 is the highest expressing FZD protein and shows the most important role in Wnt/β-catenin activation, whose expression is furthermore regulated by an adjacent lncRNA, termed lncFZD6 [62]. As the core component of Wnt/β-catenin signaling, β-catenin is regulated by ncRNAs at various levels. Lnc-β-catm was screened out as a critical modulator for Wnt/β-catenin activation via TOPFlash luciferase assay. Lnc-β-catm is highly expressed in liver CSCs and required for liver CSC self-renewal and tumor initiation. Lnc-β-catm interacts withβ-catenin and EZH2 through different regions, and they form a ternary complex depending on the scaffold role of lnc-β-catm. The association of EZH2 facilitates the methylation of β-catenin at K49, and the methylation inhibits phosphorylation and ubiquitination of β-catenin, inducing an increased β-catenin level and enhanced Wnt/β-catenin activation. Accordingly, lnc-β-catm induces β-catenin activation through posttranslational modification [63]. Similar to lnc-β-catm, lncTIC1 was also proved to be involved in β-catenin modification and decay. LncTIC1 was screened out as one of the most highly expressed lncRNAs in liver cancer and interacts with N terminal of β-catenin to suppress the phosphorylation and ubiquitination of β-catenin [64]. Asβ-catenin stability involved ncRNA, circβ-catenin functions as a decoy for GSK3β, but not participates in posttranslational modification. Circβ-catenin is mainly formed by exon regions of β-catenin and harbors protein-coding potentials to generate 370-amino acid β-catenin isoform, starting as same as WT β-catenin but stopping at a different site. β-catenin isoform binds GSK3β vigorously and releases the WT β-catenin from the degradation complex, promoting the activation of Wnt/β-catenin signaling [65]. Wnt/β-catenin target genes, including c-Myc, TCF7, CCDN1 and Axin2, are requisite for the biological function of Wnt/β-catenin, and the transcriptional process of these target genes is frequently regulated by ncRNAs. For examples, lncTCF7 is originated near from *TCF7* locus and regulates the accessibility of *TCF7* promoter *in cis* [56].

### 3.4 Notch signaling

Notch signaling is a fundamental pathway in embryonic development and morphogenesis, and its roles in tumorigenesis are controversial, depending on tumor types, Notch members, and even different experimental models. For example, Notch1 functions as a tumor suppressor in hepatocellular carcinoma, whereas Notch2 promotes liver tumorigenesis [45,66]. However, it is widely supposed that Notch signaling drives the self-renewal of CSCs, although convincing evidence is missing for many tumor types.

The crosstalk of Notch signaling and ncRNAs have been widely explored. Notch1 is one of the most frequently mutant genes in T cell acute lymphoblastic leukemia (T-ALL), and more than 50% of cases harbor active mutations of Notch1. Trimarchi et al. integrated the transcriptome and epigenome data and identified many previously undefined lncRNAs as Notch target transcripts in T-ALL [67]. LncRNA LUNAR1 is a typical Notch target lncRNA and is required for the propagation of T-ALL *in vitro* and *in vivo*, proving that lncRNAs are functional targets of Notch signaling in T-ALL [67]. Different from Notch target gene LUNAR1, NALT (Notch1 associated lncRNA in T ALL) functions upstream of Notch signaling. NALT is originated from Notch1-adjacent region and induces the transcription of Notch1 *in cis*, and thus activates Notch1 signaling [68].

### 3.5 Hedgehog signaling

Hedgehog (Hh) signaling is another critical signaling pathway in development and its hyperactivation is detectable in various tumors and CSCs. In the absence of Hh ligands, the Hh receptor Ptch interacts with Smo and promotes plasma membrane retention of Smo. Upon engagement of the Hh ligand and its receptor Ptch, Ptch is internalized and releases Smo from the plasma membrane, which then locates at the primary cilium for activation. Smo translocation causes activation of the Gli TF family to trigger the expression of downstream target genes [69].

LncHDAC2 was identified as one of critical lncRNAs in liver CSCs using transcriptomics and sphere formation functional assay. LncHDAC2 is highly expressed in liver cancer and especially liver CSCs and is essential for liver tumorigenesis and CSC self-renewal through LOF and ectopic expression assays. LncHDAC2 interacts with HDAC2 directly to recruit NURD complex to *PTCH1* promoter. LncHDAC2-dependent NURD-enrichment inhibits transcription of PTCH1 and finally promotes SMO translocation and Hedgehog activation. LncHDAC2, NURD components HDAC1, HDAC2, CHD3, and Hedgehog target gene Gli3 are positively related to HCC severity, providing the importance of lncHDAC2-Hedgehog axis in liver tumorigenesis [70]. CircIPO11, a circRNA generated from *IPO11* locus, is also involved in the activation of Hedgehog signaling. CircIPO11 is highly expressed and functionally important circRNAs in liver CSCs. CircIPO11 knockout impairs liver CSC self-renewal, liver tumor initiation, and propagation both in human cells and in mouce cells. CircIPO11 interacts with TOP1, a topoisomerase responsible for relaxing supercoiled DNA during replication and transcription. CircIPO11 recruits TOP1 to *Gli1* promoter and induces Gli1 expression, which further activates Hh signaling and promotes liver CSC self-renewal [71]. Wu et al. identified circ-SMO as protein-coding circRNAs to maintain Hh signaling activation [72]. Circ-SMO is highly expressed in brain CSCs and encoded SMO-193aa which plays a critical role in brain CSC self-renewal and Hh signaling activation. SMO-193aa interacts with and promotes cholesterol modification of SMO, and then induces the release of SMO from PTCH-induced depression state and finally causes Hh signaling activation. Moreover, circ-SMO and SMO-193aa are positively regulated by Gli1 target gene Fus, forming a feedback circle “circ-SMO→SMO-193aa→SMO→Gli1→Fus→circ-SMO” to induce Hh activation and brain CSC self-renewal [72].

### 3.6 Hippo/Yap1 signaling

Hippo/Yap1 signaling is traditionally considered a central modulator in controlling organ size, and accumulating studies have revealed its critical roles in immune regulation, tumorigenesis and tissue stem cells [73]. Yap1 is phosphorylated by Lats for inactivation upon Hippo-ON, and Yap is activated and translocates to the nucleus for target gene transcription upon Hippo-OFF. Hippo/Yap1 signaling is emerging as a fundamental pathway controlling the self-renewal of CSCs [74].

Lnc-CTHCC is a cancer-testis (CT) gene-related lncRNA and is highly expressed in liver cancer and testis. Lnc-CTHCC knockout mice, knockdown cells, and ectopic expression assays demonstrate a critical role of lnc-CTHCC in liver CSC self-renewal, propagation, metastasis, and tumorigenesis. The lnc-CTHCC interacts with hnRNP K and recruits it to *Yap1* promoter. Consequently, lnc-CTHCC drives the self-renewal of liver CSCs through Yap1 signaling [75]. The lncRBM (lncRNA for association with Brahma) is also highly expressed in liver cancer and liver CSCs and is required for liver CSC self-renewal via LOF and ectopic expression assays. The lncRBM interacts with BRM, but not BRG1, and is involved in the switch from BRM-embedded SWI/SNF to BRG1-embedded SWI/SNF, which then induces Yap1 expression through Klf4. Klf4 binds *Yap1* promoter and recruits BRG1-type SWI/SNF to drive the accessibility of *Yap1* promoter and activation of Yap signaling [49]. CircPIP5K1A is a Yap-associated circRNA vigorously expressed in osteosarcoma cells. CircPIP5K1A depletion causes impaired proliferation, enhanced apoptosis, attenuated invasion and migration, elevated E-Cadherin expression, and declined Vimentin expression. Moreover, circPIP5K1A-depleted cells show repressed self-renewal capacity, decreased CSC ratios, and stem factor expression. Harboring MiR-515-5p binding sites, circPIP5K1A serves as a sponge of MiR-515-5p to block binding between miR-515-5p and its target gene Yap1 and finally promotes Yap1 expression and activation [76].

### 3.7 Other signaling pathways

BMP signaling in CSCs is also controversial, depending on the tumor type and BMP family members. Wang et al. reported that BMPR1A (bone morphogenetic protein receptor type 1A) plays an oncogenic role in liver tumorigenesis and liver CSCs [77]. Moreover, BMPR1A expression is modulated by a conserved lncRNA, Hand2-AS in humans and lncHand2 in mice. LncRNA Hand2-AS is highly expressed in liver CSCs, and its knockout impairs the initiation, propagation, and self-renewal of liver cancer in both human cells and mice. Hand2-AS is associated with the INO80 chromatin-remodeling complex and recruits it to the *BMPR1A* promoter, which further induces BMPR1A expression and subsequent activation of BMP signaling, finally promoting the self-renewal of liver CSCs [77].

EGFR (epidermal growth factor receptor)-STAT3 signaling is a fundamental pathway in tumorigenesis and propagation, and hyperactivation of EGFR is observed in 50% of glioblastoma. Gao et al. explored peptide-coding circRNAs using RNA-seq and ribosome profiling and identified circ-E-Cad as a functional circRNA in self-renewal and tumor initiating capacity of glioblastoma CSCs [78]. Circ-E-Cad encodes a 254-amino-acid protein via multiple rounds of translation, which is named C-E-Cad. As a secretory protein, C-E-Cad interacts with EGFR CR2 domain and activates STAT3, PI3K–AKT, and MAPK–ERK pathways in glioblastoma CSCs. Serving as an EGF-independent EGFR-activator, C-E-Cad blockade dramatically enhances therapeutic effect of anti-EGFR strategy, which alone is proven ineffective for glioblastoma treatment [78].

PI3K/AKT/mTOR is also involved in cell determination and self-renewal regulation in various tumors. Circ_0007059 is downregulated in liver cancer, and its overexpression inhibits the proliferation and self-renewal of liver CSCs, and promotes their apoptosis. Circ_0007059 promotes PTEN expression by acting as a sponge of miR-421, and PTEN knockdown or AKT blockade abolishes the regulatory role of circ_0007059. Circ_0007059 finally induces mTOR activation and liver CSC self-renewal through PTEN, a typical antagonist of PI3K/AKT/mTOR signaling [79].

## 4 Regulation of ncRNAs in cancer cells

**Fig. 3:**
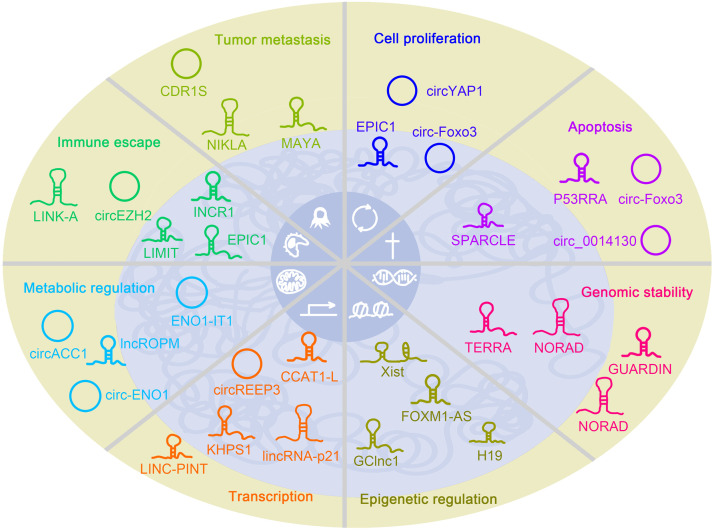
**ncRNAs in tumor development and progression.** ncRNAs are involved in indicated tumorigenesis-associated processes, including cell proliferation, apoptosis, genomic stability, epigenetic regulation, transcription of oncogenes and tumor suppressor genes, metabolic regulation, immune escape, and tumor metastasis. Typical ncRNAs are listed, and their subcellular locations are denoted.

Tumor cells harbor abnormal characteristics in various aspects, including cell proliferation, cell death, genome instability, epigenetic regulation, abnormal expression of oncogene and tumor repressor genes, metabolic disorders, immune escape, and metastasis [2,3]. Recently, ncRNAs are emerging as critical modulators that regulate these tumor hallmarks (Fig. 3).

### 4.1 Cell proliferation

Hyperactivation of cell proliferation is a typical characteristic of cancer cells and is frequently regulated by ncRNAs. Almost all ncRNAs involved in CSC self-renewal maintenance are also involved in proliferation, specifically, the proliferation of CSCs [51,70]. Here, we focus on ncRNAs that regulate the cell cycle, which is essential for cell proliferation and controlled by various regulators, including cyclins, cyclin-dependent kinases (CDKs), and cyclin-dependent kinase inhibitors (CKIs).

EPIC1 (epigenetically-induced lncRNA1) is one of 1006 lncRNAs whose promoters are hypomethylated across 6475 tumors and 455 cancer cell lines. EPIC1 promotes cell proliferation by transcriptional regulation of several cell cycle associated genes, including CDKN1A, CCND2, CDC20, and CDC45, via a c-Myc dependent manner. EPIC1 interacts with c-Myc and promotes occupancy of c-Myc to its target genes, and EPIC1 LOF impairs expression of these cell cycle associated genes. This work reveals the importance of lncRNAs in cell cycle regulation function of c-Myc and provides a new avenue for c-Myc targeting based on its interaction with EPIC1 [80]. Circ-Foxo3 is lowly expressed in various tumors and its expression inhibits cell proliferation via blockade of cell cycle entrance. Circ-Foxo3 interacts with P21 and Cdk2 simultaneously to form a ternary complex. Cdk2 combines with cyclin A or cyclin E to promote cell cycle progression, and P21 inhibits Cdk2/CCNA and Cdk2/CCNE. Circ-Foxo3-depended P21-Cdk2 interaction blocks the formation of Cdk2/CCNA and Cdk2/CCNE and thus inhibits cell cycle entrance and cell proliferation [81]. CircYAP1 has an inhibitory role in gastric tumorigenesis and cell proliferation. CircYAP1 harbors miR-367-5p binding sites and sponges miR-367-5p, which targets p27Kip1, a critical CKI for cell cycle arrest. Accordingly, circYAP1 ectopic expressing cells show decreased free miR-367-5p, increased p27Kip1, and attenuated cell proliferation [82].

### 4.2 Apoptosis

Tumor cells escape cell death during tumor initiation, propagation, and even drug treatment. To date, cell death induced by intracellular or extracellular elements is the main strategy for almost all therapeutics. Numerous types of cell death have been identified, including apoptosis, necroptosis, pyroptosis, and ferroptosis [83]. The involvement of ncRNAs in cell death regulation has been extensively explored in recent years. Here, we focus on ncRNAs in apoptosis regulation, and the readers can refer to this review for details about ncRNAs in necroptosis, pyroptosis, and ferroptosis regulation [84].

The lncRNA P53RRA is lowly expressed in multiple tumors via epigenetic DNA methylation silencing mechanism, and its ectopic expression inhibits tumor propagation and induces cell apoptosis and ferroptosis. P53RRA binds to G3BP1 through 1–187 nt of P53RRA and RRM domain of G3BP1, and their combination induces p53 release from G3BP1 complex and subsequent nuclear translocation, where p53 targets several metabolic genes to trigger cell apoptosis and ferroptosis [85]. Besides cell proliferation, circ-Foxo3 also participates in apoptosis regulation. Circ-Foxo3 interacts with MDM2 and p53 simultaneously to form a complex. Enhanced interaction of MDM2 and p53 further causes p53 ubiquitination and degradation. Furthermore, the formation of MDM2-circ-Foxo3-P53 ternary complex releases Foxo3 from Foxo3-MDM2 degradable complex, followed by increased Foxo3 expression. Finally Foxo3 target gene Puma is robustly expressed to induce cell apoptosis [86]. CircRNA Hsa_circ_0014130 is highly expressed in non-small cell lung cancer (NSCLC) and is required for tumor propagation and invasion via inhibiting apoptosis. Hsa_circ_0014130 functions as a sponge of miR-136-5p and inhibits the combination of miR-136-5p and its target gene Bcl-2. Accordingly, hsa_circ_0014130 promotes the expression of Bcl-2 via a ceRNA-dependent manner and finally inhibits cell apoptosis in NSCLC [87]. LncRNA SPARCLE (suicidal PARP-1 cleavage enhancer) was recently identified as a p53 inducible transcript that is required for p53-mediated cell apoptosis in late DNA damage response. SPARCLE inhibits DNA repair and promotes DNA-damage-induced cell apoptosis, via strong interaction with PARP-1. SPARCLE functions as a caspase-3 cofactor for PARP-1 cleavage into N-terminal PARP-1 (NT- PARP-1), which inhibits DNA repair. Thus in late DNA damage response, p53 promotes apoptosis via a SPARCLE-dependent manner [88].

### 4.3 Genome stability

Genome instability, generally including gene mutation, rearrangements, and copy number alternation, is central to tumorigenesis and used for tumor therapeutics [89]. The genome is frequently attacked by numerous risk factors from normal metabolic activities such as oxygen radicals and environmental biophysicochemical factors such as radiation. Rapid proliferation in tumorigenesis also frequently induces DNA mismatch. Serious DNA damage usually induces cell apoptosis, while moderate damage generally induces DNA repair to maintain genomic stability, with a large number of genes and ncRNAs involved. LOF of genes involved in genomic stability and DNA repair, such as p53 mutation, is a common characteristic of tumorigenesis.

LncRNA NORAD, abbreviated for Noncoding RNA Activated by DNA Damage, is a well-known lncRNA maintaining genomic stability [90]. NORAD is a highly conserved lncRNA and robustly expressed upon DNA damage, with 500–1000 copies per cell. Moreover, *NORAD* knockout or knockdown causes dramatic aneuploidy in genome-stable cells, proving a critical role of NORAD in genomic stability. However, there are at least two molecular mechanisms for NORAD in controlling genomic stability. Lee et al. obtained 5 biotinylated NORAD fragments for RNA pulldown assay and subsequent mass spectrometry, filtered the proteins that were at least 5 times enrichment in RNA pulldown eluate for all 5 fragments and finally identified the interaction of NORAD and multivalent PUMILIO proteins. NORAD harbors multiple PUMILIO proteins to sequester PUMILIO efficiently, and PUMILIO binds to and degrades mRNAs that are responsible for mitotic regulation, DNA repair, and DNA replication. Altogether, NORAD is located exclusively in the cytoplasm and maintains genomic stability via a PUMILIO-dependent manner [90]. Munschauer et al. discovered that NORAD is both in the cytoplasm and nucleus in steady-state, and undergoes a nuclear translocation stressed state. Taking advantage of RNA antisense purification (RAP) and quantitative liquid chromatography–mass spectrometry, they identified interacting proteins in live cells and showed NORAD interacts with RBMX and their interaction is required for NORAD's function in maintaining genomic stability. Moreover, NORAD and RBMX are required for the assembly of a newly-discovered complex termed NARC1 (NORAD-activated ribonucleoprotein complex 1), which exerts an essential function in maintaining genomic stability. NORAD LOF causes disassembly of NARC1 and thus triggers genomic instability [37]. However, there is no evidence to prove the importance of NORAD-RBMX interaction in NORAD-mediated genomic stability in Munschauer's research. Additionally, another work reveals that NORAD is located in cytoplasm both in the steady-state and DNA-damaging state. Moreover, a genetic rescue experiment proved that PUMILIO—NORAD binding, but not RBMX-NORAD binding, is required for NORAD's function in genomic stability regulation [91].

LncRNA TERRA (telomeric-repeat-containing RNA) originates from chromosome ends and regulates telomere maintenance and genome stability. TERRA binds telomeres and regulates telomeric state via telomerase and homology-directed DNA repair, whereas the mechanism of TERRA recruitment to telomeres is unclear. Feretzaki et al. established a reporter system and revealed the necessary and sufficient role of UUAGGG repeats in the telomeric location of TERRA. TERRA interacts with DNA recombinase RAD51 and forms R-loop with telomeric DNA via RAD51-dependent strand invasion. R-loop is generally formed by nascent RNA and its DNA template during transcription, but this work discovered a RAD51-dependent R-loop formation in telomeres without transcription, which mediates the recruitment of TERRA at telomeres to maintain genome stability [92]. GUARDIN, firstly identified as p53-inducable lncRNA, is another lncRNA required for genomic stability both in the steady-state and stressed state. Increased GUARDIN expression was observed in p53 ectopically expressing cells, as well as p53-WT colon tumors compared to p53-mutant tumors. GUARDIN LOF induces attenuated cell proliferation and decreased genomic integrity, and enhances cytotoxicity of genotoxic stress. GUARDIN harbors eight miR-23a binding sites and functions as miR-23a sponge to sequester microRNA-23a in *TRF2* mRNA decay, and thus prevent chromosome end-to-end fusion via a TRF2-dependent manner. Serving as a RNA scaffold, GUARDIN also promotes the interaction of BRCA1 and BARD1 to sustain BRCA1 stability. Collectly, GUARDIN functions downstream of p53 for genome stability, serving as a ceRNA and scaffold RNA [93].

### 4.4 Epigenetic regulation

As a hallmark of cancer, epigenetic disorders largely contribute to tumorigenesis, and ncRNAs themselves are typical epigenetic elements [3]. Recently, a large number of ncRNAs have been identified as modulators of other epigenetic elements, such as X-inactivation, DNA methylation, histone modifications, and RNA modifications.

As a long lncRNA of 17 kb in length, Xist is a key modulator in X-inactivation via various mechanisms, including epigenetic regulation. Taking advantage of RNA antisense purification, McHugh et al. identified Xist-interacting proteins in live cells and revealed that Xist interacts with SHARP (spen family transcriptional repressor), LBR and SAF-A. SHARP further recruits the SMRT corepressor to activate HDAC3. SHARP and HDAC3 are required for Xist-mediated transcriptional silencing and Pol II exclusion, as well as PCR2 recruitment across X chromosome [94]. Oncofetal lncRNA H19 exerts critical functions in tumorigenesis and metastasis. Zhou et al. revealed a genome-wide alternation of DNA methylation upon H19 lncRNA depletion. H19 lncRNA interacts with and inactivates SAHH, which is required for SAH hydrolysis. As a product of SAM-dependent methyltransferases, SAH is a feedback inhibitor of methyltransferases. Consistently, H19 lncRNA depletion activates SAHH, decreases SAH, and activates SAM-dependent methyltransferases across genome wide [95]. LncRNA GClnc1 is highly expressed in gastric cancer and its expression levels are related to tumor size, metastasis, and prognosis. GClnc1 LOF and ectopic expression show the critical role of GClnc1 in gastric cancer cell propagation and metastasis. As a modular scaffold, GClnc1 binds to WDR5, a key component of histone methyltransferase, and HAT2A, a histone acetyltransferase. The combination of GClnc1 changes the chromatin-binding pattern of WDR5 and HAT2A genome-wide, altering the histone modification pattern, and thus regulating the transcription of a substantial subset of genes, including a functional target gene SOD2 [96]. FOXM1-AS is a *FOXM1*-locus distributed lncRNA and modulates N6-methyladenosine of nascent FOXM1 transcript. FOXM1-AS promotes the interaction of nascent FOXM1 transcript and m^6^A demethylase ALKBH5, which decreases N6-methyladenosine levels of nascent FOXM1 transcript. FOXM1-AS-induced m6A demethylation promotes splicing and expression of pre-FOXM1 through enhancing the interaction between nascent FOXM1 transcript and HuR and finally induces FOXM1 expression and tumorigenicity of glioblastoma CSCs [97].

### 4.5 Transcriptional regulation of oncogenes and tumor suppressors

Oncogenes and tumor suppressor genes are deeply involved in tumorigenesis, and their expression needs to be precisely regulated at various levels, including transcriptional regulation by ncRNAs.

The oncogene c-Myc is regulated by ncRNAs at various levels, such as lncRNA CCAT1-L in c-Myc transcription [98] and lncRNA PVT1 in c-Myc transcription [99] and stability [36]. CCAT1-L is highly expressed in human colorectal tumors and regulates c-Myc expression *in cis*. Ectopic expression of CCAT1-L through plasmid transfection cannot localize to normal nuclear sites, whereas *in cis* overexpression of CCAT1-L through TALEN-mediated genomic insertion causes CRC propagation. CCAT1-L is required for the formation of long-range interactions between the *c-Myc* promoter and its upstream enhancer element. CCAT1-L interacts with CTCF, promotes the binding of CTCF to chromatin looping, and modulates the chromatin state at the *c-Myc* promoter, finally inducing transcription of the oncogene c-Myc [98]. The *PVT1* gene is frequently copy-number gained in tumors, and the consequent PVT1 overexpression promotes the protein stability of c-Myc [36]. In contrast to the oncogenic role of PVT1, genome-scale CRISPRi screening revealed that knockdown of PVT1 with sgRNA targeting the *PVT1* TSS region promotes cell growth [100]. The tumor-suppressor role of the *PVT1* promoter was confirmed by an *in vitro* cell growth competition assay and an *in vivo* subcutaneous xenograft assay. Mechanistically, the *PVT1* promoter inhibits c-Myc transcription via promoter-enhancer competition but not the *PVT1* transcript. The *PVT1* promoter and *MYC* promoter compete for engagement with their enhancers, and thus, the *PVT1* promoter inhibits *MYC* promoter–enhancer interaction and subsequent *MYC* transcription. The tumor-suppressor function of the *PVT1* promoter is also consistent with its recurrent translocations and deletions in human cancers [99]. The divergent function of the *PVT1* promoter and *PVT1* transcript in c-Myc expression reveals the complexity of the crosstalk between lncRNAs and oncogenes.

As a well-known tumor repressor gene, p53 functions by targeting several protein-coding genes, including p21. Huarte et al. revealed that lincRNA-p21, a lincRNA originating upstream near the *p21* locus, is also a target gene for p53 activation in two independent tumor models. LincRNA-p21 interacts with hnRNP-K and alters its genomic localization, regulating hundreds of genes enriched by p53. LincRNA-p21 and hnRNP-K co-repress p53-reponsive genes [101]. RNC-seq (ribosome nascent-chain complex-bound RNA sequencing) was used for translatome sequencing, and potential coding circRNAs were identified, including a circRNA formed by exon 2 of LINC-PINT. Circular LINC-PINT encodes an 87-aa peptide, which dramatically inhibits the proliferation and self-renewal of glioblastoma. Moreover, 87-aa peptide interacts directly with PAF1 complex and inhibits transcriptional elongation of multiple oncogenes, including CPEB1, CCND1, SOX2, c-Myc, and so on [102]. CircREEP3 is highly expressed in colorectal cancer and is required for tumorigenesis and metastasis through oncogene FKBP10. CircREEP3 recruits CHD7 to *FKBP10* promoter to enhance its transcription [103]. LncRNA KHPS1 binds the enhancer region upstream oncogene SPHK1 to form triple-helical RNA-DNA-DNA structure and recruits p300 complex to activate *SPHK* enhancer, promoting generation of enhancer RNA (eRNA-Sphk1). eRNA-Sphk1, in turn, evicts CTCF, which is traditionally regarded as a positive regulator for enhancer-promoter interaction but insulates *SPHK1* enhancer from *SPHK1* promoter. Accordingly, LncRNA KHPS1 exerts its role through oncogene SPHK1, inducing *SPHK1* promoter activation via eRNA-Sphk1-dependent CTCF excluding [104].

### 4.6 Metabolic regulation

Accumulating evidence indicates that tumorigenesis itself is a kind of metabolic disease, and abnormal metabolic regulation plays a key role in tumorigenesis and emerges as an optical target for tumor treatment [105]. Tumor cells prefer glycolysis to oxidative phosphorylation, which is termed the Warburg effect, to fuel the materials required for rapid cell proliferation and acclimate to low O_2_, low pH, and low nutrient conditions. In addition to carbohydrate metabolism, lipid metabolism, and protein metabolism are also altered in tumorigenesis. Accumulating data prove that ncRNAs are critical modulators of metabolic dysregulation [106].

Enolase 1 (ENO1), a glycolysis enzyme, plays an oncogenic role in various tumors. Hong et al. [107] and Zhou et al. [108] identified ncRNAs regulating ENO1 at transcription level and mRNA level, respectively. Hong et al. show that lncRNA ENO1-IT1 (enolase1-intronic transcript 1) is a target of *F. nucleatum*, a typical CRC-related microbe [107]. *F. nucleatum* induces ENO1-IT1 expression via enhancing combination of transcription factor SP1 and *ENO1-IT1* promoter. ENO1-IT1, in turn, alters histone modification pattern of *ENO1* promoter via KAT7 histone acetyltransferase. Hence, *F. nucleatum* abundance is correlated with glucose metabolism, at least partially through ENO1-IT1-mediated ENO1 expression [107]. Zhou et al. identified circ-ENO1 as a ceRNA for *ENO1* mRNA stability in lung adenocarcinoma [108]. Circ-ENO1 leads to tumor propagation and metastasis via glycolysis. Circ-ENO1 harbors miR-22-3p binding site and functions as miR-22-3p sponge to block combination of miR-22-3p and *ENO1* mRNA, thus promoting stability of *ENO1* mRNA and glycolysis activity [108].

To identify functional circRNAs in lipid metabolism, Li et al. cross-referenced the validated circRNA list and lipid metabolism genes and selected circACC1 as a functional circRNA in colon cancer, whose depletion led to significant lipid accumulation. CircACC1 was upregulated by serum deprivation in a c-Jun-dependent manner and played critical roles in metabolic reprogramming and tumor propagation. CircACC1 interacted directly with AMPK β and γ subunits, promoted the assembly, stability, and activity of AMPK holoenzyme, and drove glycolysis and fatty acid oxidation via an AMPK-dependent manner [109]. Another ncRNA involved in lipid metabolism is lncROPM (a regulator of phospholipid metabolism). LncPORM was highly expressed in breast CSCs and drove the self-renewal of breast CSCs *in vivo* and *in vitro*. LncPORM bound the 3’-UTR of *PLA2G16* and enhanced the mRNA stability of PLA2G16, an lncROPM adjacent gene involved in phospholipid metabolism. Increased arachidonic acid-induced by LncPORM-PLA2G16 axis activated PI3K/AKT, Wnt/β-catenin, and Hippo/YAP signaling, and further drove breast CSC self-renewal [110].

### 4.7 Immune escape

Immune surveillance is a major obstacle against tumorigenesis [2,3]. Tumor cells adopt diverse strategies to avoid attacking immune cells, such as increasing the expression of immune checkpoints, reducing antigenicity with MHC downregulation, secreting immunosuppressive molecules, and recruiting immunosuppressive cells. Meanwhile, tumor cell-intrinsic ncRNAs have emerged as critical modulators of immune escape.

NcRNAs involved in MHC-based antigen presentation have been extensively investigated. LINK-A (long intergenic non-coding RNA for kinase activation) was originally discovered as a modulator of normoxic HIF1α stabilization [111] and is also involved in immune escape. Taking advantage of large-scale bioinformatic analysis and validation with clinical samples, Hu et al. showed that LINK-A expression is related to immunosuppression and immunotherapy resistance in breast cancer. LINK-A promotes the interaction of PtdIns(3,4,5)P3 and inhibitory GPCR, inhibits PKA-mediated phosphorylation of TRIM71, and enhances ubiquitination-mediated degradation of Tb, p53, and antigen peptide-loading complex (PLC). Consequently, LINK-A induces breast tumorigenesis via immune escaping as well as tumor-intrinsic pathways. LINK-A transgenic mice generate mammary gland tumors similar to human TNBCs at genomic, transcript, and metabolic levels. Moreover, targeting LINK-A prevents tumorigenesis and progression, and sensitizes breast tumor cells to immunotherapy [112]. The lncRNA EPIC1 (epigenetically induced lincRNA 1) is another modulator of tumor cell antigen presentation. Guo et al. developed a lincRNA-based immune response (LIMER) using TCGA database and identified EPIC1 as a potent modulator for tumor immune in multiple tumor types. EPIC1 interacts with EZH2 and regulates the expression of IFNGR1, TAP1/2, ERAP1/2, and MHC-I, leading to an impaired presentation of tumor antigen. EPIC1 ectopic expression causes immune evasion and resistance to immunotherapy, and inhibition of EPIC1-EZH2 emerges as a potential strategy for immunotherapy [113]. Li et al. divide TCGA and SKCM melanoma into hot and cold tumors and show LIMIT (lncRNA inducing MHC-I and immunogenicity of tumor) is enriched in hot tumors. LIMIT is stimulated by IFNγ and cis-activated transcription of LIMIT adjacent gene GBP2, which further activates HSF1 via disrupting HSF1-HSP90 interaction. HSF targets and activates transcription of MHC-I machinery, including HLA-ABC, TAP1, HSPA5, and CALR. LIMIT LOF and cis-activation by CRISPRa strategy induce an impaired and enhanced presentation of tumor antigen. Clinically, LIMIT-GBP-HSF1 axis is related to MHC-I and checkpoint blockade response [114].

Surface markers involved in “tumor-immune” interaction are also regulated by ncRNAs, contributing to immune escape. PD-L1, an extensively investigated molecule involved in tumor immune, is exclusively regulated by IFNγ according to traditional research. Mineoet al. identified INCR1 (IFN-stimulated non-coding RNA 1) as a typical PD-L1 modulator. *INCR1* locus is between *JAK2* and *PD-L1* gene location and can be efficiently induced by IFNγ. LncRNA INCR1 interacts with HNRNPH1 and blocks its binding to PD-L1 and JAK2 transcripts, thus promoting PD-L1 and JAK2 expression. INCR1 knockdown inhibits PD-L1 expression and promotes therapeutic effect of CAR T cells [115]. Circular EZH2 is highly expressed in glioblastoma cells and encodes EZH2-92aa, which binds *MICA/B* promoters to suppress their expression and also represses ULBP transcription via stabilizing EZH2. Consequently, NKG2D ligands, containing MICA/B and ULBP, are down-regulated by EZH2-92aa in glioblastoma, which accounts for immune escape of glioblastoma to NK cells in clinical treatment. Moreover, EZH2-92aa silenced cells show impaired NK escape and synergize with anti-PD1 therapy [116].

### 4.8 Tumor metastasis

Metastasis is a major obstruction for tumor therapy and is controlled by diverse pathways, such as the TGFβ–ZEB1/ZEB2 pathway and NF-κB pathway. NcRNAs have emerged as critical modulators of metastasis [117].

CDR1as is traditionally regarded as an atypical circRNA with an unknown linear counterpart; meanwhile, CDR1as functions as a miR-7 sponge and is one of the first discovered circRNAs serving as a miRNA sponge [29]. Hanniford et al. revealed that CDR1as is generated from upstream LINC00632, and functions as a repressor of invasion and metastasis in melanoma cells [118]. Unexpectedly, CDR1as exerts its role in a miR-7-independent manner but via IGF2BP3, an oncogene and a reader of m^6^A [118]. LncRNA MAYA (MST1/2-antagonizing lncRNA for YAP activation) was screened from a human Lincode siRNA library using a TEAD-driven luciferase reporter assay. MAYA is required for LLGL2-MAYA-NSUN6 axis regulation, which is recruited by ROR1-mediated p-HER3 Tyr1307 to methylate Hippo/MST1 at Ly59. Consequently, MAYA promotes crosstalk between ROR1–HER3 and the Hippo–YAP pathway and thus induces bone metastasis of breast cancer cells [119]. NKILA (NF-KappaB Interacting LncRNA) is another metastasis-associated lncRNA. NKILA is stimulated by several inflammatory cytokines in a NF-kB-dependent manner, whereas its ectopic expression inhibits NF-kB activation. NKILA interacts with the NF-κB/IκB complex through two NKILA motifs and two P65 sites, and blocks phosphorylation of IκB by masking IKK phosphorylation sites, thus impairing NF-κB activation. Consequently, NKILA inhibits breast cancer metastasis and patients with low NKILA expression show poor survival with metastasis tendency [120].

## 5 Regulation of ncRNAs in tumor environment

Tumor cells and CSCs are influenced by a complicated tumor environment (niche for CSCs), including diverse immune cells, stromal cells, and extracellular matrix [51]. Tumorigenesis is a result of the mutual interaction of tumor and niche cells, and ncRNAs are emerging as critical modulators in tumor environment regulation (Table 1).

**Table 1:** **The ncRNAs in the regulation of tumor environment.** Typical ncRNAs for tumor environment regulation are shown, including ncRNA name, tumor type, location, “tumorniche” cross-talk, molecular mechanism, and reference.

ncRNA	Tumor type	Location	Tumor-niche interaction	Molecular mechanism	Ref
lncRNA-MUF	Liver cancer	Cancer cell	MSCs drive liver CSC self-renewal via lncRNA-MUF	LncRNA-MUF binds ANXA2 to activate Wnt/β-catenin pathway	94
circUBAP2	Liver cancer	Cancer cell	CAFs induce circUBAP2 expression to drive liver cancer propagation and metastasis	circUBAP2 sponge miR-4766 to drive downstream IL-17/IL-1β expression	95
NKILA	NSCLC & breast cancer	CTLs/Th1s	Activation-induced cell death of CTLs and Th1s diminishes anti-tumor immunity	NKILA is triggered by TCR- induced calcium influx and drives AICD by NF-κB blockade	96
Lnc-EGFR	Liver cancer	TILs	The differentiation of iTreg to Treg cells inhibits CTL activity and promotes tumorigenesis	Lnc-EGFR interacts with and stabilizes EGFR to promote Foxp3 expression via AP-1	97
IRENA	Breast cancer	TAMs	IFNs-driven TAM switch promotes antitumor immunity and chemoresistance	IRENA interacts with PKR to induce its dimerization and phosphorylaiton for NF-kB activation	98
MALAT1	Thyroid cancer	TAMs	TAMs secret FGF2 to promote the proliferation and metastasis of thyroid cancer	MALAT1 drives FGF2 expression and secretion	99
circFARP1	Pancreatic cancer	CAFs	CAFs secret LIF to activate LIF- STAT3 pathway for gemcitabine resistance	circFARP1 interacts with and stabilizes CAV1, and drives LIF mRNA stability via miR-660-3p	103
circCUL2	Pancreatic cancer	CAFs	CAFs secret IL-6 to trigger STAT3 activation in pancreatic cancer for propagation	circCUL2 functions as miR-203 sponge to drive MyD88-NFkB activation and IL-6 secretion	104
HISLA	Breast cancer	Extracellular vesicles	TAMs-released EVs promote glycolysis and apoptotic resistance of breast cancer	HISLA interacts with PHD2 to stabilize HIF1α for glycolysis and apoptotic resistance	105
circHIF1A	Breast cancer	Exosomes	Exosomes from gypoxic CAFs promote CSC self-renewal	circHIF1A sponges miR-580-5p to promote CD44 expression	106
LINC00659	Colorectal cancer	Exosomes	CAF-derived exosomes drive colorectal cancer propagation	LINC00659 interacts with miR- 342-3p for ANXA2 expression	107
H19	Colorectal cancer	Exosomes	CAF-derived exosomes promote CSC self-renewal	H19 sponges miR-141 to activate β-catenin pathway	108
LNMAT2	Bladder cancer	Exosomes	Tumor-secreted exosomes promote lymphangiogenesis	LNMAT2 binds to PROX1 promoter and drives its expression in HLECs	109
SNHG16	Breast cancer	Exosomes	Tumor-secreted exosomes droive a switch from γδ1 to T_reg_ cells for immunosuppression	SNHG16 interacts with miR-16-5p to promote Smad5 expression and CD73 activation	110

### 5.1 Tumor-intrinsic ncRNAs for tumor environment regulation

Tumor cells receive various biophysicochemical signals from the tumor environment, and tumor-intrinsic ncRNAs are involved in this process. Immune escape is a typical response for niche immune cells, and tumor cell intrinsic ncRNAs involved in immune escape have been described in Section 4.7. In this section, we focus on the ncRNAs involved in crosstalk between tumor cells and stromal cells, which are broadly defined as nontumor and nonimmune cells in the tumor bulk.

Yan et al. revealed that mesenchymal stem cells (MSCs) promote self-renewal of metastasis of liver cancer through lncRNA-MUF (MSC upregulated factor) [121]. A co-culture of liver cancer cells and MSCs was established and MSC-stimulation increases liver CSC ratios and enhances sphere formation, tumor initiation, propagation, and metastasis capacities. MSC alters lncRNA expression profile in cancer cells, and lncRNA-MUF is dramatically up-regulated in liver cancer cells upon MSC treatment. LncRNA-MUF binds ANXA2 to activate Wnt/β-catenin signaling and sponges miR-34a to activate Snail1 and collectively promotes hepatocarcinogenesis and metastasis [121]. CAF (Cancer-associated fibroblast) secretes CXCL11 to induce liver cancer propagation and metastasis through circUBAP2, which is dramatically up-regulated upon CAF induction and functions as miR-4766 sponge to block inhibitory role of miR-4766 on IFIT1/IFTI3. Consequently, circUBAP2 maintains IFIT1/IFTI3 expression, which then induces downstream IL-17/IL-1β expression and secretion to promote propagation and metastasis of liver cancer [122].

### 5.2 Regulation of ncRNAs in immune cells

As typical tumor environment cells, immune cells are extensively involved in tumor initiation and tumor therapy. Escaping from immune surveillance is a basic requisite for tumor initiation and propagation [123], and tumor immunotherapy provides vast promise for various tumors, especially for hematologic malignancies. NcRNAs emerge as pivotal modulators in tumor-associated immune cells and regulate immune responses at various levels.

NKILA, a typical ncRNA involved in NF-κB activation, is highly expressed in tumor-specific CTLs and Th1s, but not Th2s and Treg cells and is responsible for specific AICD (activation-induced cell death) of CTLs and Th1s. TCR-induced calcium influx activates the *NKILA* promoter by removing deacetylase, and NKILA sensitizes CTLs and Th1s to AICD by inhibiting NF-κB activity. NKILA-depleted CTLs show attenuated AICD and thus inhibit the *in vivo* propagation of breast cancer and non-small cell lung cancer. Clinically, NKILA expression in CTLs and Th1s is related to their apoptosis and patient survival [124]. Lnc-EGFR is highly expressed in tumor-infiltrating lymphocytes, and its expression is positively related to Treg content, Foxp3 expression, and tumor size, but negatively related to IFN-γ expression. Lnc-EGFR is highly expressed in Treg cells and promotes differentiation from iTreg to Treg cells. Lnc-EGFR interacts with EGFR, promotes EGFR stability by inhibiting its ubiquitination, and then activates the EGFR/AP-1/NF-AT1 axis to promote Foxp3 expression. Through Treg differentiation, Lnc-EGFR inhibits CTL activity and drives liver carcinogenesis [125].

Tumor associated macrophages (TAMs) can be classified into an anti-inflammatory phenotype (M2) that promotes immunosuppression and tumor propagation, and a proinflammatory phenotype (M1) that mediates antitumor immunity. Liu et al. revealed that chemotherapy-induced IFNs induce TAM phenotype switching from M2 to M1, which drives antitumor immunity via Jak-STAT1-mediated cytokines but also initiates chemoresistance via NF-kB-mediated cytokines [126]. Interestingly, lncRNA IRENA (IFN-responsive nuclear factor-κB activator) is a central mediator of IFN-induced NF-kB activation in TAMs. IRENA is induced by the Jak/STAT pathway upon IFN stimulation and interacts with PKR to trigger its dimerization and anto- phosphorylation, which further triggers NF-kB activation. IRENA LOF doesn't affect enhanced antitumor immunity mediated by IFN, but diminishes the chemoresistance effect, demonstrating that IRENA blockade is an optimal strategy to avoid chemoresistance while retaining antitumor immunity [126]. LncRNA-MALAT1 (metastasis-associated lung adenocarcinoma transcript 1) is highly expressed in TAMs and promotes the secretion of FGF2, thus promoting the proliferation, migration, and invasion of thyroid cancer cells. LncRNA-MALAT1 knockdown in TAMs inhibits the propagation of thyroid cancer cells, and FGF2 overexpression rescues the inhibitory effects of MALAT1, indicating that lncRNA-MALAT1 exerts its role in an FGF2-dependent manner [127].

ILCs (innate lymphoid cells) are recently defined immune cell types, and participate in microbial clearance, parasitic exclusion, and tumor immunity [128]. NcRNAs emerge as critical modulators in ILC differentiation, maintenance, and functionality. LncKdm2b is highly expressed in ILC3s and sustains ILC3 maintenance in a Zfp292-dependent manner. LncKdm2b interacts with Bptf and recruits the NURF complex to the *Zfp292* promoter for transcriptional activation, which is required for ILC3 maintenance [129]. CircZbtb20 is also highly expressed in ILC3s, and its deletion decreases ILC3 numbers and impairs ILC3 function. CircZbtb20 promotes the interaction of Alkbh5 and Nr4a1, which further decreases the N6-methyladenosine level of *Nr4a1* mRNA. Nr4a1, in turn, promotes Notch signaling activation and ILC3 maintenance [130].

### 5.3 Regulation of ncRNAs in stromal cells

Stromal cells, broadly defined as nontumor and nonimmune cells in tumors, are a key structural component of tumors and extensively contribute to tumorigenesis, and the functions of stromal cell-intrinsic ncRNAs in tumorigenesis have been defined recently.

As a typical kind of stromal cells, CAFs (cancer-associated fibroblasts) are involved in gemcitabine resistance of pancreatic cancer [131]. CircFARP1 is highly expressed in CAFs compared to normal fibroblasts, and induces gemcitabine resistance via LIF secretion. CircFARP1 interacts with CAV1, blocks the interaction of CAV1 with E3 ligase ZDRF1, and thus promotes protein stability of CAV1. CAV1 is a major integral protein of caveolae to account for LIF secretion. Meanwhile, circFARP1 also sponges miR-660-3p to block miR-660-3p-mediated *LIF* mRNA decay, leading to a stabilized *LIF* mRNA. Altogether, circFARP1 promotes CAF-mediated LIF secretion and thus induces gemcitabine resistance of pancreatic cancer via LIF-STAT3 pathway [131]. CircCUL2 is another circRNA highly expressed in CAFs and mediates propagation of pancreatic cancer. CircCUL2 functions as miR-203 sponge and blocks the inhibitory role of miR-203 on *MyD88* mRNA, thus promoting MyD88 expression. MyD88 hyperactivation causes NFkB activation and IL-6 secretion in CAFs, and IL-6 further triggers STAT3 signaling activation in pancreatic cancer [132].

### 5.4 Extracellular ncRNAs

Generally, ncRNAs themselves have no secretory signal and cannot be secreted directly, while the existence and functionality of extracellular ncRNAs have been extensively investigated in recent years [133,134]. Extracellular ncRNAs can be secreted from niche cells or tumor cells, which are involved in “tumor-niche” interactions. These ncRNAs generally reside in vesicles, the critical mediator in cell–cell contact-independent communications.

Niche cells regulate biological functionality of tumor cells via various mechanisms, including the secretion of ncRNA-containing vehicles. Chen et al. revealed an lncRNA-based interaction between TAMs and breast cancer cells. HISLA (HIF-1α-stabilizing long noncoding RNA) is highly expressed in TAMs upon lactate stimulation from glycolytic tumor cells [135]. HISLA-containing extracellular vesicles (EVs) can be released from TAMs, and HISLA promotes glycolysis and apoptotic resistance of breast cancer cells through interacting with PHD2, which mediates a quick hydroxylation and ubiquitination-induced decay of HIF1α. HISLA inhibits the interaction of HIF1α and PHD2, thus promoting the stability of HIF1α and increasing glycolysis product lactate. HISLA-mediated positive feedback between breast cancer cells and TAMs enhances aerobic glycolysis and chemoresistance of breast cancer [135]. Another HIF1α-associated extracellular ncRNA is circHIF1A, which was screened out by differential circRNA-seq with exosomes derived from hypoxic CAFs and normoxic CAFs. CircHIF1A is increased in exosomes from hypoxic CAFs and promotes self-renewal of breast CSCs. CircHIF1A functions as a miR-580-5p sponge and promotes the expression of CD44, a widely-accepted CSC marker involved in self-renewal [136]. LINC00659 is highly expressed in CAFs compared to normal fibroblasts and is transferred from CAFs to colorectal cancer cells via exosomes. LINC00659 interacts with miR-342-3p to promote ANXA2 expression and induces propagation, invasion, migration, and EMT of colorectal cancer cells [134]. Similarly, another lncRNA termed H19 is also expressed in CAFs and functions in colorectal cancer cells via an exosome-dependent manner. As a ceRNA for miRNA, H19 binds to miR-141 and blocks the inhibitory role of miR-141 in β-catenin pathway. Consequently, exosomal H19 promotes self-renewal of colorectal CSCs via β-catenin pathway [137].

On the contrary, tumor cells also generate ncRNA-embedded extracellular exosomes for niche remodeling. Bladder cancer undergoes LN (lymph node) metastasis mainly through VEGF-C–dependent manner. Chen et al. revealed an lncRNA-mediated LN metastasis via VEGF-C–independent manner [133]. The lncRNA LNMAT2 (lymph node metastasis-associated transcript 2) is highly expressed in bladder cancer and loads into tumor-secreted exosomes via interacting with hnRNPA2B1. Exosomal LNMAT2, in turn, is internalized into HLECs (human lymphatic endothelial cells) and binds to *PROX1* promoter via forming DNA-RNA triplex. LNMAT2-dependent PROX1 activation induces lymphangiogenesis and lymphatic metastasis [133]. SNHG16 is another lncRNA in cancer cell-derived exosomes. SNHG16 is highly expressed in breast cancer cells and loaded into exosomes and then internalized into γδ1 T cells, most abundant tumor-infiltrating lymphocytes (TILs) in breast cancer. SNHG16 interacts with miR-16-5p and blocks the binding of miR-16-5p and *Smad5* mRNA, consequently promoting expression of Smad5, which further initiates TGF-β-mediated CD73 expression. CD73^+^ γδ1 T cells are predominant Treg population in breast cancer and function as an immunosuppressor via an adenosine-mediated pathway [138].

## 6 The ncRNA-based diagnoses and therapies

### 6.1 Highly expressed ncRNAs as potential tumor biomarkers

Compared with protein-coding genes, lncRNAs display enhanced tissue-specific expression patterns [139] and circRNAs are enriched in exosomes and more stable than linear mRNAs [140]. These characteristics make lncRNAs and circRNAs optimal cancer biomarker candidates, and many tumor-specific ncRNAs have been identified for tumor diagnosis.

Based on the Cox regression model on ccRCC (clear cell renal cell carcinoma) RNA-seq data from TCGA (The Cancer Genome Atlas) and LASSO (least absolute shrinkage and selection operation), Qu et al. built an RCClnc4 classifier based on four lncRNAs: ENSG00000255774, ENSG00000248323, ENSG00000260911, and ENSG00000231666 [141]. In Both the training set and validation set, RCClnc4 was significantly used to classify ccRCC patients into high-risk and low-risk groups and could be used to predict risk stratification for ccRCC patients[141]. LncRNA H19 is another biomarker released from tumor cells and enriched in the peripheral blood of gastric cancer patients. Plasma levels of H19 can distinguish gastric cancer patients, even early-stage gastric cancer patients Thus, H19 will be used as a biomarker for the diagnosis of gastric cancer and early cancer screening [142]. CircRNA circOSBPL10 is another ncRNA upregulated in gastric cancer and can be used as a survival marker of gastric cancer patients [143]. CircRNA F-circEA, originating from the *EML4-ALK* fusion gene, is enriched in the plasma of *EML4-ALK*-positive non-small cell lung cancer (NSCLC), serving as an additional “liquid biopsy” biomarker for NSCLC [144].

### 6.2 Functional ncRNAs and drug-resistance

Drug resistance is a major challenge for tumor therapies and is largely related to cancer mortality [145]. Many protein-coding genes have been identified in drug-resistance, and recently, ncRNAs have been defined as critical modulators in this process.

To identify functional lncRNAs in cytarabine resistance, Bester et al. analyzed drug response-associated lncRNAs using the Cancer Cell Line Encyclopedia (CCLE) and Cancer Target Discovery and Development (CTD2) databases, and performed CRISPRa screening with 14 days of Ara-C treatment [146]. Computational and functional analyses identified many lncRNAs and protein-coding genes involved in cytarabine resistance, including GAS6-AS2. GAS6-AS2 is originated from *GAS6* locus and initiates GAS6 expression to activate GAS6/TAM signaling via cis-regulation. GAS6-AS2 also promotes AXL transcription via DNMT-mediated methylation at *AXL* promoter. Altogether, GAS6-AS2 was screened from dual protein-coding and non-coding integrated CRISPRa screening (DICaS), and functions as upstream activator of GAS/AXL signaling axis [146]. CircRNA-SORE (sorafenib-resistant) is a circRNA highly expressed in sorafenib resistant HCC cells and largely accounts for sorafenib resistance. Moreover, circRNA-SORE can be transported by exosomes and spread sorafenib-resistant capacity among HCC cells. CircRNA-SORE interacts with YBX1 and prevents PRP19-mediated degradation of YBX1, and blockade of circRNA-SORE/YBX1 signaling dramatically improves therapeutic effect of sorafenib [147]. Besides chemotherapeutics, ncRNAs are also involved in the resistance of immunotherapies. CircFGFR1 is highly expressed in NSCLC tumors and induces propagation, metastasis, and immune evasion of NSCLC cells. CircFGFR1 combines miR-381-3p and functions as miR-381-3p sponge to promote expression of CXCR4, a target gene of miR-381-3p. CircFGFR1-CXCR4 signaling promotes immunotherapeutic resistance of NSCLC [148].

### 6.3 Oncogenic ncRNAs as therapeutic targets

ncRNAs are traditionally targeted by small-interfering RNA (siRNA), antisense oligonucleotides (ASO), or locked nucleic acid (LNA), and accumulating ncRNAs have been proven to be optimal targets in mouse xenograft models. Taking advantage of nascent RNA capture sequencing, Ali et al. investigated lncRNAs enriched in S-phase and revealed SCAT7 (RP11-465N4.4) as a functional lncRNA in multiple cancer types [149]. Then, they depleted SCAT7 using siRNA and LNA in cell lines or patient-derived cells and confirmed the potential therapeutic role of SCAT7 [149]. AC104041.1, a lncRNA highly expressed in HNSCC (head and neck squamous carcinoma) and functions as ceRNA of miR-6817-3p, promotes Wnt/β-catenin activation and CSC maintenance via enhancing expression of miR-6817-3p target gene Wnt2. Treatment with AC104041.1 ASOs inhibits tumor growth and CSC self-renewal, and its therapeutic effect is enhanced upon the combination with salinomycin, an inhibitor of Wnt/β-catenin signaling [150]. NEAT1_1 (3.7 kb) and NEAT1_2 (23 kb) are two NEAT1 isoforms and play opposite roles in propagation of neuroblastoma cells. NEAT1_1 harbors a growth-promoting role whereas NEAT1_2 has a growth-inhibiting role via a paraspeckle-dependent manner. NEAT1_1 is generated by efficient cleavage and polyadenylation at the expense of NEAT1_2, and a higher NEAT1_1: NEAT1_2 ratio is detected in neuroblastoma. ASO-mediated sterical blockade of NEAT1_1 polyadenylation decreases NEAT1_1 level, increases NEAT1_2/paraspeckles and de-regulates NEAT1_1: NEAT1_2 ratio, thus exerts anti-tumor effect via activation of differentiation pathways, which are usually repressed in high-risk neuroblastoma [151].

Almost all drugs on the market target one of approximately 700 disease-associated genes, and there are no drugs targeting ncRNAs yet. There are many more ncRNAs than protein-coding genes, which will open new avenues for drug discovery if ncRNAs are targetable. However, compared to protein targets generally sustaining stable conformations, ncRNAs usually adopt several conformations, and it's a big challenge to explore the structures of ncRNAs. Aguilar et al. purified RepA domain of Xist and screened RepA binding components from 50,000 Merck's compounds via LC–MS based ALIS (automated ligand identification system), and X22 was screened out as RepA partner and used for analogs optimization [152]. As a finally defined RepA-binding component, X1 inhibits the interaction of RepA and PRC2, decreases histone H3K27 tri-methylation, and blocks X-chromosome inactivation role of Xist. X-ray scattering analysis reveals that RepA adopts several conformations but favors one structure in solution, and X1 binding further stabilizes RepA conformation [152]. This proof of concept research demonstrates that ncRNAs are targetable and also opens a new avenue for targeting oncogenic ncRNAs.

### 6.4 Delivery of tumor inhibitory ncRNAs

Despite great significance, it is a major challenge to deliver inhibitory RNAs for tumor targeting. Various biological materials have been developed and recently nanoparticle-based gene delivery systems have shown outstanding advantages in the COVID-19 mRNA vaccines mRNA-1273 and BNT162b2, for which lipid nanoparticles are used for COVID-19 mRNA delivery [153]. Diverse nanoparticles have also been designed to target tumors, and the delivery of inhibitory ncRNAs might be a new avenue for tumor targeting.

Circ-1073 is expressed at low levels in breast cancer cells, and its overexpression dramatically inhibits breast cancer cell proliferation, metastasis, and epithelial-mesenchymal transition, and promotes apoptosis. Micropoly nanoparticles containing circ-1073 are used for breast cancer treatment, leading to impaired xenograft tumor growth [154]. LncRNA SLERCC (Specific Low Expression in RCC) is lowly expressed in renal cell carcinoma via DNMT3A-mediated *SLERCC* promoter hypermethylation and its overexpression inhibits Wnt/β-catenin signaling and RCC metastasis. Taking advantage of the efficient inhibitory role of SLERCC, Plasmid-SLERCC@PDA@MUC12 nanoparticles were constructed and tested *in vivo* and *in vitro*, and SLERCC-delivery nanoparticle dramatically inhibits the growth of RCC metastases *in vivo* [155]. Lin et al. established a metastasis model of breast cancer cells and confirmed the predominant role of breast cancer stem cells in tumor metastasis [156]. CircRGPD6 is lowly expressed in metastatic cells and inhibits metastasis through miR-26b/YAF2 axis. Moreover, TV-circRGPD6 nanoparticle delivers circRGPD6 into breast tumor to inhibit tumor propagation and metastasis [156]. Zhao et al. designed mitochondria-targeting nanoparticles for mitochondrial delivery, and circRNA SCAR was delivered to mitochondria, where it interacts with ATP5B [22]. As we know, the subcellular location of biomacromolecules is critical for their roles, and mitochondria are difficult to be targeted. Delivery of mitochondria-located circRNA provides a new avenue for organelle-specific delivery of ncRNAs.

## 7 Perspective and conclusion

Here, we review the roles of ncRNAs in cancer stem cells, tumor cells, and tumor environmental cells. We also discuss the potential applications of ncRNAs in tumor diagnosis and therapy. In the future, advanced technologies and RNA sequencing methods, including RIC-seq [157], LACE-seq [158], single-cell CAS-seq [159], CIRI-long and nanopore sequencing [160], are needed to facilitate the investigation of ncRNA biogenesis and interactomics. Moreover, cutting-edge technology needs to be invented for exploring the interactions of ncRNAs-ncRNAs, ncRNAs-DNAs, and ncRNAs-RBPs. For instance, CRISPR/Cas9 mediated ncRNA KO mice provide an optimal model for *in vivo* investigation. CRISPR-based screening, including the CRISPRi Non-Coding Library [100] and RfxCas13d/BSJ-gRNA system [161], is used to genomically identify functional ncRNAs. NcRNA-based diagnosis and therapy have emerged as a promising avenue for cancer research, however, clinical applications of ncRNAs are still challenging and no successful examples have been developed. The difficult translation of RNA-based therapies into the clinic is hampered by issues related to the specificity, delivery, and tolerability of ncRNAs [162]. Successful applications of RNA-based therapies require interdisciplinary approaches and technological advances. We believe that ncRNA-based tumor diagnosis and therapy will take place soon.

## Declaration of competing interest

The authors declare that they have no conflicts of interest in this work.
